# Supplementary material for: Transcriptome Profile Alterations with Carbon Nanotubes, Quantum Dots, and Silver Nanoparticles: A Review
Source: Genes (Basel). 2021 May 23;12(6):794. doi: 10.3390/genes12060794 (PMC8224664; doi:10.3390/genes12060794)
Supplement: Supplementary file 1 [file genes-12-00794-s001.zip › genes-1179744-supplementary.pdf]

Table S1. Summary and comparison of findings for CNTs.

CNT		
**Common Uses**	**Positive Findings**	**Negative Findings**
cancer therapy	Structure of CNT appealing for drug delivery [1].	Microarray shows differentially expressed genes related to cancer when treated with MWCNT [2].
bone cell proliferation	anti-bacterial treatment by altered protein and ribosome expression, down regulated genes associated with glucose metabolism, DNA damage and oxidative stress [3].	Lung cancer biomarker upregulated when treated with MWCNT [4].<br><br>Lung disease related to SWCNT [5].
novel battery technologies	alternative microbial agent for food borne pathogens by differential gene expression related to membrane proteins and increased ROS production [6].	biomarker for human lung diseases: LC7A1 and SLC22A5 were downregulated in all mice and human tissue, blood and cell analyses. Affects: human primary hypertension, cardiovascular diseases, encephalopathy, cardiomyopathy, cardiomegaly, metabolic derangement, hypoglycemia, and muscle weakness [1, 7]
photovoltaics		Increased expression of ddit3 genes result in inflammatory response and increased ER stress when treated with MWCNT [8].
nano based transitioners		Increase in spindle disruption, abnormal mitotic spindles, and aneuploid chromosome number with the increased doses of MWCNTs [9].

Table S2. Summary and comparison of findings for QDs.

QD		
**Common Uses**	**Positive Findings**	**Negative Findings**
therapeutic targeting	QD-treated bacteria became more sensitive to polymyxin B which make them great candidates for adjuvant therapies for bacterial infections [10].	QDs negatively impact functions including transport, biosynthesis, and metabolism in *E. coli* [10].
treatment of cancer	Can efficiently deliver drugs to specific molecular targets, including cancer cells, at subcellular levels [11].	Pharmacokinetic of QD toxicity is not fully understood and could have potentially negative impacts to our health [11].

biological imaging	CdSe/ZnS QDs could be an effective alternative anticancer drug. With RNA-seq, they found HeLa cells up-regulated anti-tumorigenic functions [12].	Hazards encountered with QDs are much more complex than limitations created by common probes [11].
drug delivery system	InP/ZnS QDs could be a potential anticancer drug. With RNA-seq, they observed pro-apoptotic processes and control over motility in HeLa cells [13].	Hazards encountered with QDs are much more complex than limitations created by traditional delivery systems [11].
	QDs linked to some ligands have shown to emit brighter and be more photostable than organic dyes [11].	
	QDs have the potential to treat/diagnosis cancer with site-directed delivery because of their tunable fluorescence and modifiable surfaces for targeting [11].	

Table S3. Summary and comparison of findings for AgNPs.

**AgNP**		
**Common Uses**	**Positive Findings**	**Negative Findings**
Antibacterial treatment [14, 15]	Green synthesis for eco-friendly production [14, 16-19].	Essential genes linked to AgNP induced toxicity in yeast (met9, sfh1, and peg1) [20].
Water disinfection [21]	Anti-bacterial treatment by down-regulating TCA cycle genes (aceF, gadB) [22].	Upregulation of target genes MT and GST for understanding AgNP toxicity in yeast as it relates to chemogenetic screening [23].
Biofouling control [24]	Downregulation of biofilm formation genes in S. epidermidis (icaA and icaR) and S. aureus (fnbA and fnbB) [25].	Decreased viability in S. cerevisiae as a result of disrupted ribosome function and cell wall organization [26].
Agriculture and livestock treatments (Lee et al, Siddiqi et al, Kalinska et al)	Regulation of Hfq function in S. aureus as antibacterial mechanism [27].	Repression of cell survival genes in mouse fibroblast resulting in increased apoptosis [28].
Medical diagnostics (Lee et al)	Aflatoxin biosynthesis genes AFB1 and omt-A inhibited in A. flavus after AgNP treatment [29].	Increased expression of genes relevant to Alzheimers (GSS, CYCL12, MARCO) after AgNP exposure in mice neural cells [30].
	Inhibition of pathogenic melanin production in fungi by downregulation of PKS1 and SCD1 [31].	Altered genetic expression leading to increased susceptibility to carcinogens [32].

Increased expression of proapoptotic genes (p53, p21, Cyt C, Bid, Bax, Bak) in cervical cancer cells as potential anti-cancer treatment [33].

1. Snyder-Talkington, B.N., et al., *Multi-walled carbon nanotube-induced gene expression in vitro: concordance with in vivo studies.* Toxicology, 2015. **328**: p. 66-74.
2. Gao, J., et al., *A novel clinically translatable fluorescent nanoparticle for targeted molecular imaging of tumors in living subjects.* Nano Lett, 2012. **12**(1): p. 281-6.
3. Liu, D., Y. Mao, and L. Ding, *Carbon nanotubes as antimicrobial agents for water disinfection and pathogen control.* Journal of Water and Health, 2018.
4. Pacurari, M., et al., *Multi-walled carbon nanotube-induced gene expression in the mouse lung: association with lung pathology.* Toxicol Appl Pharmacol, 2011. **255**(1): p. 18-31.
5. Fan, J., et al., *Inhibition of autophagy contributes to the toxicity of cadmium telluride quantum dots in Saccharomyces cerevisiae.* Int J Nanomedicine, 2016. **11**: p. 3371-83.
6. Hussain, S., et al., *One-pot fabrication of high-quality InP/ZnS (core/shell) quantum dots and their application to cellular imaging.* Chemphyschem, 2009. **10**(9-10): p. 1466-70.
7. Chang, S., et al., *Cytotoxicity, cytokine release and ER stress-autophagy gene expression in endothelial cells and alveolar-endothelial co-culture exposed to pristine and carboxylated multi-walled carbon nanotubes.* Ecotoxicol Environ Saf, 2018. **161**: p. 569-577.
8. Long, J., et al., *The adverse vascular effects of multi-walled carbon nanotubes (MWCNTs) to human vein endothelial cells (HUVECs) in vitro: role of length of MWCNTs.* J Nanobiotechnology, 2017. **15**(1): p. 80.
9. Siegrist, K.J., et al., *Genotoxicity of multi-walled carbon nanotubes at occupationally relevant doses.* Part Fibre Toxicol, 2014. **11**: p. 6.
10. Monras, J.P., et al., *Microarray analysis of the Escherichia coli response to CdTe-GSH Quantum Dots: understanding the bacterial toxicity of semiconductor nanoparticles.* BMC Genomics, 2014. **15**: p. 1099.
11. Ghaderi, S., B. Ramesh, and A.M. Seifalian, *Fluorescence nanoparticles "quantum dots" as drug delivery system and their toxicity: a review.* J Drug Target, 2011. **19**(7): p. 475-86.
12. Hens, B., et al., *The Future of Anticancer Drugs: A Cytotoxicity Assessment Study of CdSe/ZnS Quantum Dots.* Nanotheranostics, 2020. **19**(38): p. 20.
13. Davenport, V., et al., *An assessment of InP/ZnS as potential anti-cancer therapy: Quantum dot treatment induces stress on HeLa cells* 2020.
14. Samuggam, S., et al., *Green Synthesis and Characterization of Silver Nanoparticles Using Spondias mombin Extract and Their Antimicrobial Activity against Biofilm-Producing Bacteria.* Molecules, 2021. **26**(9).
15. Ghodake, G., et al., *Extracellular Synthesis and Characterization of Silver Nanoparticles-Antibacterial Activity against Multidrug-Resistant Bacterial Strains.* Nanomaterials (Basel), 2020. **10**(2).
16. Irfan, M., et al., *Ecofriendly development of electrospun antibacterial membranes loaded with silver nanoparticles.* Journal of Industrial Textiles, 2021.
17. Salayova, A., et al., *Green Synthesis of Silver Nanoparticles with Antibacterial Activity Using Various Medicinal Plant Extracts: Morphology and Antibacterial Efficacy.* Nanomaterials (Basel), 2021. **11**(4).
18. Tan, L.V., T. Tran, and V.D. Thi, *Biosynthesis of Silver Nanoparticles from Bacillus licheniformis TT01 Isolated from Quail Manure Collected in Vietnam.* Processes 2021. **9**(4).
19. Mahiuddin, M., P. Saha, and B. Ochiai, *Green Synthesis and Catalytic Activity of Silver Nanoparticles Based on Piper chaba Stem Extracts.* Nanomaterials (Basel), 2020. **10**(9).

20. Lee, A.R., et al., *Editor's Highlight: A Genome-wide Screening of Target Genes Against Silver Nanoparticles in Fission Yeast.* Toxicol Sci, 2018. **161**(1): p. 171-185.
21. Das, S., et al., *Disinfection of the Water Borne Pathogens Escherichia coli and Staphylococcus aureus by Solar Photocatalysis Using Sonochemically Synthesized Reusable Ag@ZnO Core-Shell Nanoparticles.* Int J Environ Res Public Health, 2017. **14**(7).
22. Ashmore, D., et al., *Evaluation of E. coli inhibition by plain and polymer-coated silver nanoparticles.* Rev Inst Med Trop Sao Paulo, 2018. **60**: p. e18.
23. Sillapawattana, P., M.C. Gruhlke, and A. Schaffer, *Effect of silver nanoparticles on the standard soil arthropod Folsomia candida (Collembola) and the eukaryote model organism Saccharomyces cerevisiae.* Environ Sci Eur, 2016. **28**(1): p. 27.
24. Dong, X., et al., *Thiol-Affinity Immobilization of Casein-Coated Silver Nanoparticles on Polymeric Membranes for Biofouling Control.* Polymers (Basel), 2019. **11**(12).
25. Wang, J., et al., *Silver-nanoparticles-modified biomaterial surface resistant to staphylococcus: new insight into the antimicrobial action of silver.* Sci Rep, 2016. **6**: p. 32699.
26. Horstmann, C., et al., *Transcriptome Profile Alteration with Cadmium Selenide/Zinc Sulfide Quantum Dots in Saccharomyces cerevisiae.* Biomolecules, 2019. **9**(11).
27. Tian, H., et al., *Antibacterial activity of silver nanoparticles target sara through srna-teg49, a key mediator of hfq, in staphylococcus aureus.* Int J Clin Exp Med, 2015. **8**(4): p. 5794-9.
28. Gurunathan, S., et al., *Cytotoxicity and Transcriptomic Analysis of Silver Nanoparticles in Mouse Embryonic Fibroblast Cells.* Int J Mol Sci, 2018. **19**(11).
29. Deabes, M.M., et al., *Impact of Silver Nanoparticles on Gene Expression in Aspergillus Flavus Producer Aflatoxin B1.* Open Access Maced J Med Sci, 2018. **6**(4): p. 600-605.
30. Huang, C.L., et al., *Silver nanoparticles affect on gene expression of inflammatory and neurodegenerative responses in mouse brain neural cells.* Environ Res, 2015. **136**: p. 253-63.
31. Mishra, S. and H.B. Singh, *Silver nanoparticles mediated altered gene expression of melanin biosynthesis genes in Bipolaris sorokiniana.* Microbiol Res, 2015. **172**: p. 16-8.
32. Nallanthighal, S., J.P. Heiserman, and D.J. Cheon, *The Role of the Extracellular Matrix in Cancer Stemness.* Front Cell Dev Biol, 2019. **7**: p. 86.
33. Yuan, Y.G., et al., *Silver Nanoparticles Potentiates Cytotoxicity and Apoptotic Potential of Camptothecin in Human Cervical Cancer Cells.* Oxid Med Cell Longev, 2018. **2018**: p. 6121328.
